# Surgical Management of Massive Labial Edema in a Gravid Preeclamptic Diabetic

**DOI:** 10.1155/2014/935267

**Published:** 2014-10-12

**Authors:** Jennifer S. Lindsey, James E. DeVente

**Affiliations:** ^1^Division of Reproductive Endocrinology and Infertility, Department of Obstetrics, Gynecology and Reproductive Medicine, Brigham and Women's Hospital and Harvard Medical School, Boston, MA 02115, USA; ^2^Department of Obstetrics and Gynecology, Brody School of Medicine, East Carolina University, 600 Moye Boulevard, Greenville, NC 27834, USA

## Abstract

*Background*. Massive labial edema is a rare complication during pregnancy that can jeopardize vaginal delivery, as well as leading to maternal and fetal morbidity. It can be related to systemic pathologies, but has been commonly associated with preeclampsia and diabetes. This increased and sometimes longstanding pressure may result in a “labial compartment syndrome” leading to microvascular damage and tissue necrosis if not resolved in a timely fashion. *Case*. Massive labial edema was treated first conservatively and then surgically in a gravid diabetic patient with severe preeclampsia. Immediately after Cesarean section, the labial compartment syndrome was relieved surgically and resolved rapidly. *Conclusion*. When conservative attempts at management of labial edema fail, or rapid resolution is critical to maternal and fetal outcome, surgical alternatives should be considered.

## 1. Introduction

Massive labial edema is an unusual complication of pregnancy that may occur due to an underlying systemic pathology but has also been associated with preeclampsia. It has the potential to interfere with vaginal delivery and compromise tissue integrity and has been associated with maternal mortality postpartum [1, 2]. We present a surgical alternative to conservative management of massive labial edema in a gravid, preeclamptic patient.

## 2. Case Presentation

A 21-year-old woman, gravida 2, para 1, at 32-week, gestation was transferred to our facility after initiating uterine contractions one week prior and was evaluated for uterine contractions, elevated blood sugars, and massive labial edema. Her cervical dilatation was unchanging at 1 cm and her blood sugars were labile during admission. Her history was remarkable for a recent urinary tract infection, genital warts, preeclampsia during first pregnancy, and Class C diabetes poorly managed with multiple episodes of diabetic ketoacidosis over the previous decade. Due to the impressive degree of the labial minora edema, which was approximately 6 cm × 10 cm, shiny, and translucent, the patient was evaluated for infection, autoimmune and connective tissue disorders, abdominal mass, preeclampsia, nutritional deficiencies, vascular abnormalities, and pregnancy-related causation. She was treated with tight glucose management via EndoTool, diphenhydramine, ice packs, and morphine.

Overnight on hospital day two, the edema worsened and extended to the labia majora as well as legs, arms, abdomen, and face. The patient developed tachypnea and mild respiratory distress congruent with pulmonary edema, although oxygen saturation was 98–100%. She was hypertensive with a blood pressure of 164/94 mmHg. Total serum protein and albumin were low, at 4.9 g/dL and 2.8 g/dL, respectively. At this point, seizure prophylaxis with magnesium sulfate was initiated due to concern about preeclampsia. Conservative monitoring was continued as pulmonary edema improved. Late that evening, pulmonary edema worsened and the patient experienced increased difficulty in breathing, as well as a marked increase in labial pain and edema.

**Figure 1:**
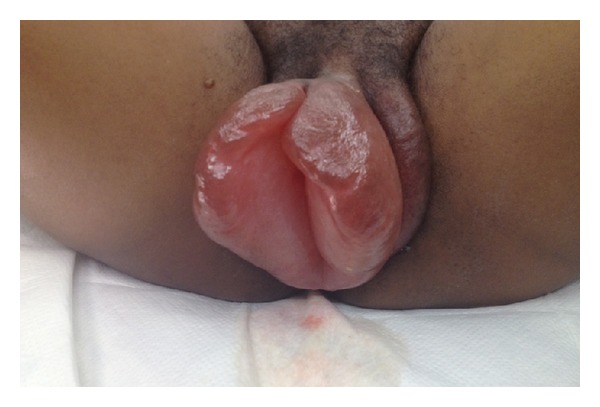
Massive edema of the labia minora prior to incisions and drainage.

Based upon clinical evidence of pulmonary edema, severe range blood pressures, and 24-hour urine protein of 760 mg, the patient was determined to have preeclampsia with severe features. The patient's laboratory findings were negative for HELLP syndrome. Induction of labor was impossible due to massive labial edema. The patient was consented for primary low transverse Cesarean section. Cesarean delivery was uneventful, and after skin closure, attention was turned to the massive labial edema (Figure 1). Two, 1.5 cm linear incisions were placed on the medial aspects of the labial minora. Copious amounts of clear transudate immediately flowed from the incisions, and expression released further fluid. The incisions were left open to allow additional drainage.

Over the subsequent 10 hours, the patient continued to have difficulty oxygenating (oxygen saturations between 87 and 97%) secondary to her pulmonary edema. An echocardiogram was performed in the immediate postpartum period that demonstrated a structurally normal heart with normal biventricular size and systolic function. She had worsening of her edema in both lower extremities; however her labial edema improved markedly. Nine hours later, the massive labial edema was much improved and the patient could again sit upright comfortably with no vaginal complaints. By postpartum day 5, the labial edema was completely resolved.

## 3. Discussion

While edema is common in gravid women, present in 8 of 10 patients, it is typically found in the lower extremities, face, or hands. Massive edema confined to the labia has only been reported in a handful of antepartum cases. It may present unilaterally or bilaterally, as in the case presented. Our patient's condition seemed to be a sequela of her preeclampsia and diabetes. Other known causes include infectious, neoplastic, or inflammatory disorders, as well as trauma or tocolysis [3–7].

Historically, reports on massive labial edema suggest that it may be used as a diagnostic tool to indicate the severity of the patient's preeclampsia [6, 8]. During pregnancy, the significant increase in blood volume, blood pressure, and vascular permeability, coupled with compression of the inferior vena cava by the growing uterus, can cause edema. While the exact pathophysiology of preeclampsia remains poorly understood, the disease is characterized by both systemic and localized vascular endothelial dysfunction. This is demonstrated focally in the pulmonary edema, cerebral edema, and hepatic capsule rupture and failure, all of which are significant risks of preeclampsia [9]. Additionally, decreased renal function is also implicated in that it affects serum protein concentrations and salt retention [10]. Other relevant mechanisms include venous congestion, narrowing of venules, which increases pressure in capillary beds [11], and a defective venoarteriolar reflex [12]. Together, many of these mechanisms may result in both systemic and localized edema, as vasculature leaks fluid into the interstitial spaces. For supine or seated patients, this can be restricted to the labia as it is the lowest point in the body. Severe labial edema has been suggested to signal severe progression of preeclampsia [6]. Preeclampsia, which complicates 3–9% of all pregnancies in developed countries [13], is life-threatening to both mother and infant and is a leading cause of both maternal and neonatal morbidity and mortality worldwide.

Conservative management of labial edema is the most commonly reported form of treatment and concludes with spontaneous resolution postpartum [6, 14, 15]. Efforts to alleviate the symptoms also include Trendelenburg positioning [3, 16, 17], placement of ice packs [18], correction of nutrient deficiencies [19], and water immersion therapy [5]. However, this methodology requires continued surveillance and often results in prolonged discomfort for the patient. Conservative management is not appropriate in situations where maternal or fetal health is at risk, as is the case with severe preeclamptic patients. Furthermore, these types of management do not adequately prevent tissue deterioration and necrosis due to compartment syndrome, if the edema is ongoing.

Few physicians have reported employing surgical management of massive labial edema [7, 17]. As was demonstrated in our patient, a small incision to the affected area can relieve fluid and pressure, facilitating blood flow to the edematous region. This can result in a comparatively rapid resolution of the condition. In our patient, sitz baths and ice packs did not improve the edema. We recommend that when conservative management fails or rapid resolution of labial edema is critical to avoiding poor maternal outcome, surgical alternatives should be considered. After delivery, we chose to proceed in this manner, placing incisions and expressing the edematous fluid, because the skin overlaying the labia showed signs of necrosis and deterioration. Prolonged edema might result in lasting tissue and microvasculature damage. Additionally, rapid resolution was desired to alleviate the prolonged and severe discomfort of the patient.

With the incidence of preeclampsia increasing in the Unites States, specifically a 25% rise in the last 20 years [20], the incidence of massive labial edema will likely parallel this rise. In situations where conservative management proves to be ineffective, or where there is a suspicion of “labial compartment syndrome,” immediate surgical management shows rapid resolution of this condition and a favorable delivery outcome.
